# Saturated Fatty Acid Increases Lung Macrophages and Augments House Dust Mite-Induced Airway Inflammation in Mice Fed with High-Fat Diet

**DOI:** 10.1007/s10753-017-0550-4

**Published:** 2017-04-01

**Authors:** Hiroki Tashiro, Koichiro Takahashi, Hironori Sadamatsu, Go Kato, Keigo Kurata, Shinya Kimura, Naoko Sueoka-Aragane

**Affiliations:** 1 Division of Hematology, Respiratory Medicine and Oncology, Department of Internal Medicine, Faculty of Medicine, Saga University, 5-1-1 Nabeshima, Saga, 849-8501 Japan; 2 Institute of Tokyo Environmental Allergy, Tokyo, Japan

**Keywords:** bronchial asthma, obesity, high-fat diet, house dust mite, saturated fatty acid, macrophages

## Abstract

Obesity is one of the phenotypes of severe asthma, which is considered to be a heterogeneous syndrome; however, its interaction with airway inflammation is not fully understood. The aim of this study was to clarify the role of saturated fatty acids in augmenting airway inflammation induced by house dust mite (HDM) in obesity. Subjects were Balb/c mice fed a high-fat diet (HFD) for 10 weeks, followed by sensitization and exposure to HDM. Subjects were also administered palmitic acid (PA) for 4 weeks with concurrent sensitization and exposure to HDM. Airway inflammation was assessed by quantifying the amount of inflammatory cells in bronchoalveolar lavage (BAL) and airway resistance was measured. *In vitro*, lipopolysaccharide (LPS)-primed macrophages were stimulated by PA. The amount of monocyte chemoattractant protein-1 (MCP-1), interleukin-1β (IL-1β), and tumor necrosis factor α (TNF-α) was examined in the supernatant. Compared to normal chow mice, HFD mice underwent significant increases in body weight; increases in number of lung macrophages, including circulating monocytes and alveolar macrophages; and increases in bronchoalveolar lavage fluid (BALF) total cell count, including neutrophils but not eosinophils, after HDM sensitization and exposure. *In vitro*, PA induced MCP-1 and augmented LPS-primed production of IL-1β and TNF-α in macrophages. Among HDM mice that were administered PA, there was an increase BALF total cell count, including neutrophils but not eosinophils, compared to vehicle mice. In conclusion, saturated fatty acid increased the number of lung macrophages and augmented HDM-induced neutrophilic airway inflammation in a HFD mouse model.

## INTRODUCTION

Bronchial asthma is a common respiratory disease that involves eosinophilic airway inflammation induced by sensitization and exposure to antigens, such as house dust mite (HDM) [14, 23]. Inhaled corticosteroid (ICS), which is the principal medication for the treatment of asthma, has contributed to disease control and reduction of mortality for the past 20 years [47]. However, 5% to 10% of cases that are refractory to standard treatment are identified as severe asthma [8, 27, 35]. Severe asthma is characterized by uncontrolled symptoms, frequent exacerbations, airflow limitation, and airway inflammation [28, 46]. As a result, patients with severe asthma need higher cost of medical treatment than those with mild asthma [1, 2]. Severe asthma is considered to be a heterogeneous syndrome that has features of early-onset atopic factor, late-onset eosinophilic airway inflammation, neutrophilic airway inflammation, and obesity [35, 46]. Basic as well as clinical research has shown that obesity is an important phenotype of severe asthma [3, 20, 34, 38]. In addition, asthmatic patients who are obese do not respond as well to ICS as patients with normal body mass index (BMI) [4, 33, 39]. In fact, weight loss was shown to improve airway hyperresponsiveness (AHR) and symptom control in obese asthmatic patients [11, 12]. These studies implied that the extent of obesity and the severity of asthma are closely related; however, the interaction between obesity and the pathogenesis of asthma, including airway inflammation, is not fully understood.

Obesity itself is considered an inflammatory disease [45] that is associated with other low-grade systemic inflammatory diseases, such as metabolic syndrome, type 2 diabetes, non-alcoholic fatty liver, and cardiovascular disease [17, 43]. In previous studies, animal models were administered a high-fat diet (HFD) to induce obesity so that the interaction between obesity and inflammation could be analyzed [15, 18, 22]. Overconsumption of saturated fatty acids (SFA), which compose a HFD, was discovered to be a risk factor for obesity-related diseases [13, 37]. SFA induces inflammatory molecules, such as tumor necrosis factor α (TNF-α), interleukin (IL)-1β, IL-6, monocyte chemoattractant protein-1 (MCP-1), and macrophage inhibitory factor through toll-like receptor 4 (TLR4) [36, 44, 52], and regulates organ inflammation through macrophage recruitment [9, 50]. According to these data, increased amount of SFA in obese individuals would lead to inflammation in various organs.

In the present study, an increased number of lung macrophages were observed in a HFD mouse model. HDM-induced mice with augmented neutrophilic airway inflammation and AHR were found to have elevated levels of IL-17A and macrophage inflammatory protein 2 (MIP2) after receiving a HFD for 10 weeks. Palmitic acid (PA), which is the main SFA component of HFD, directly induced inflammatory cytokine and chemokine production from macrophages. Finally, we demonstrated that similar to administration of HFD, administration of PA to mice increased the number of lung macrophages and augmented HDM-induced neutrophilic airway inflammation and AHR. To the best of our knowledge, this was the first report that demonstrated SFA-augmented pathogenesis of asthma in an obese mouse model; this observation was associated with lung macrophages, which are likewise considered to enhance the mechanism of obese asthma.

## MATERIALS AND METHODS

### Allergen and Chemicals

HDM extracts from *Dermatophagoides farinae* (Der f) were purchased from ITEA Inc. (Tokyo, Japan). PA (Sigma-Aldrich, Saint Louis, MO, USA) was dissolved in 50% ethanol at 60 °C to yield a 50-mM stock concentration, which was kept at −20 °C. PA was diluted to the appropriate concentration using 1% fatty acid-free bovine serum albumin (BSA) at 37 °C. The endotoxin level in the PA solution was less than the detection limit of 0.0015 EU/ml by the assay kit (Limulus ES-2, Wako, Japan).

### Mice

Female BALB/c mice (Japan SLC Inc.; Hamamatsu, Japan) aged 3–6 weeks were kept at the Saga University animal facility under specific pathogen-free conditions. Animal experiments were undertaken following the guidelines for care and use of experimental animals by the Japanese Association for Laboratory Animals Science (1987) and were approved by the Saga University Animal Care and Use Committee.

### Administration of High-Fat Diet and Palmitic Acid

Starting at 3 weeks of age, female mice were fed with either normal chow or an HFD for 10 weeks. The HFD (D12492; Research Diets Inc., New Brunswick, NJ) provided 60% of energy in the form of fat. At the age of 6 weeks, BSA or 50-μM (150 μl) palmitate–BSA complex was administered by intraperitoneal injection five times per week for 4 weeks. Body weight was measured every week.

### Protocol for House Dust Mite-Induced Airway Inflammation in Mice Administered with High-Fat Diet or Palmitic Acid

In the HFD model, mice aged 3 weeks were fed normal chow or HFD for total 10 weeks. After 7 weeks of HFD intake, mice were sensitized by intranasal administration of 25 μg HDM or phosphate-buffered saline (PBS) once a week for 3 weeks. At 10 weeks of HFD intake, mice were exposed to continuous intranasal administration of 5 μg HDM or PBS for 3 days. In the PA mouse model aged 6 weeks, 50 μM (150 μl) of palmitate–BSA complex or a BSA vehicle was administered by intraperitoneal injection five times per week for 4 weeks. Sensitization was done by intranasal administration of 25 μg HDM or a vehicle on days 2, 9, and 16. Exposure was carried out by intranasal administration of 5 μg HDM or a vehicle on days 23, 24, and 25. On days with simultaneous PA and HDM administration, PA was given 30 min before HDM inoculation. For all these models, mice were euthanized by intraperitoneal injection of sodium pentobarbital 24 h after the final exposure. Bronchoalveolar lavage fluid (BALF) and lung tissue were collected for further analyses.

### Isolation of Single Cells from Lung Tissue

Peripheral lung tissue was cut into small pieces then transferred through a 70-μm mesh before processing in a digestion buffer that included deoxyribonuclease I (Invitrogen, Waltham, MA) and collagenase type 2 (Worthington Inc., Lakewood, NJ). The remaining red cells were lysed using BD Pharm Lysis (BD Biosciences, San Jose, CA) to obtain single-cell suspensions.

### Flow Cytometry

Single-cell suspensions were pre-incubated with FcγR-specific blocking mAb and washed before staining. Cells were stained with CD11b, CD11c, CD45, and Ly6c (eBioscience, San Diego, CA) before collection on a flow cytometer (FACS Aria 2; BD Bioscience, Franklin Lakes, NJ) and analysis by FlowJo 8.3.3 software (Tree Star, Ashland, OR).

### Collection of Bronchoalveolar Lavage Fluid

BALF samples were collected, as described previously [21, 40]. Briefly, a 20-G tube was inserted in the trachea, followed by two times of lung lavage with 1 ml of saline. The cell suspension was centrifuged at 100×*g* for 5 min at 4 °C. The total number of cells was counted using a hemocytometer. Cytospin samples were prepared from the cell suspension. Cell differentiation was determined by counting at least 300 leukocytes in samples stained with Diff-Quik (Siemens, Germany).

### Airway Hyperresponsiveness to Methacholine

Briefly, mice were anesthetized with pentobarbital before insertion of an 18-G metal needle into an exposed trachea, which was connected to a forced oscillation technique (flexiVent system; SCIREQ Inc., Montreal, Canada). Next, their lungs were inflated to a pressure of 30 cmH_2_O; baseline recordings were obtained using a single frequency (2.5 Hz, 1.2 s; Snapshot-150) and a broadband low frequency (1–20.5 Hz, 3 s; Quick-Prime-3). The mice were then exposed to an aerosol of PBS. All parameters calculated from both test signals were recorded alternately every 10 s for 3 min. Finally, two deep lung inflations were given. The above protocol was repeated for five times more with aerosols containing sequentially increasing concentrations of 0.1, 1.0, 10, 20, and 50 mg/ml methacholine (Sigma-Aldrich, Japan).

### Hematoxylin–Eosin and Periodic Acid-Schiff Histology Examination

Histologic examination was performed, as previously reported [16]. Lungs were fixed with 10% neutral-buffered formalin (Wako, Japan) and embedded in paraffin. Lung sections were stained with hematoxylin and eosin (H&E) and periodic acid-Schiff (PAS).

### Preparation of Lung Homogenates

After BAL, the left lung was isolated and homogenized in 50-mM Tris-buffered saline (pH 7.4) containing 1 mM ethylenediaminetetraacetic acid, 1 mM phenylmethylsulfonyl fluoride, 1 μg/ml aprotinin, 1 μg/ml leupeptin, 1 mM Na_3_VO_4_, and 1 mM NaF. The lung homogenates were centrifuged at 10,000×*g* for 15 min; supernatants were collected and stored at −80 °C until needed.

### Quantification of Cytokines Using Enzyme-Linked Immunosorbent Assay

IL-13, TNF-α, IL-1β, IL-17A, MCP-1, and MIP2 were measured using enzyme-linked immunosorbent assay **(**ELISA) Kits (R&D Systems Inc., Minneapolis, MN), according to the manufacturers’ instructions. All samples were tested in duplicate.

### Cell Culture of RAW 264.7, Bone Marrow Macrophages, and Peritoneal Macrophages

RAW 264.7 was grown in an RPMI 1640 medium containing 10% fetal calf serum (FCS). Bone marrow (BM) cells were isolated from BALB/c mice, as previously reported [41], and were suspended at 1.0 × 10^6^ cells/ml in RPMI 1640 medium supplemented with 10% FCS. The cells were cultured in the presence of 10 ng/ml recombinant murine macrophage colony-stimulating factor (M-CSF) (R&D Systems Inc., Minneapolis, MN) at 37 °C in a humidified atmosphere containing 5% carbon dioxide for 6 days. On day 6, cells were harvested and cultured as BM-derived macrophages (BMMs). To obtain fresh peritoneal macrophages, mice were injected intraperitoneally with 1 ml thioglycollate (3%). After 4 days, peritoneal fluid was obtained by lavage with 10 ml PBS. The fluid was centrifuged to isolate peritoneal macrophages, which were re-suspended in RPMI 1640 medium. These cells were cultured at a density of 1 × 10^6^ cells in RPMI 1640 containing FCS and were stimulated as indicated in Fig. 3. RNA was isolated after 8 h and the supernatant was analyzed by ELISA after 24 h.

### RNA Extraction and Quantitative PCR

RNA was extracted from RAW 264.7 using the RNeasy Protect Mini Kit (QIAGEN, Netherlands); assessed by quantity and quality using a NanoDrop 1000A spectrophotometer (NanoDrop Products, Wilmington, DE, USA); and was reverse transcribed to cDNA. Taqman gene expression assays were used to detect TLR4 (Mm00445273-m1 Tlr4) and 18S RNA (Mm03928990-g1 Rn18s). Messenger RNA expression levels were standardized using 18S RNA expression.

### Statistical Analysis

Data were presented as mean ± standard deviation (SD). Differences between two groups were analyzed by Student’s t test. Multiple comparisons of continuous variables were analyzed using one-way analysis of variance, followed by a *post hoc* Tukey–Kramer test for multiple groups. Significance was set at a *p* value of 0.05.

## RESULTS

### High-Fat Diet Increased Body Weight and Lung Macrophages but Not Airway Inflammation and Hyperresponsiveness

To clarify the interaction between obesity and asthma, we initially focused on the lung cell population of HFD mice (Fig. 1a). The body weight of HFD mice significantly increased compared with that of normal chow mice (Fig. 1b). The appearance of HFD mice is shown in Fig. 1c. To identify the lung cell population in HFD mice, we examined single-cell suspensions by flow cytometry (Fig. 1d). Lung macrophages were classified as recruited monocytes from the systemic circulation (circulating monocytes) or resident macrophages (alveolar macrophages). In the analysis, circulating monocytes and alveolar macrophages were significantly increased in the lungs of HFD mice compared with normal chow mice (Fig. 1e, f). Circulating monocytes were characterized by the surface markers CD45^+^; CD11c^−^; CD11b^+^; and Ly6c^+^; whereas as alveolar macrophages were CD45^+^; CD11c^+^; and CD11b^−^. The total cell count and differential count in BALF were not different between HFD mice and normal chow mice. AHR also showed no difference between these groups (data not shown). These results suggested that HFD increased the number of lung macrophages, including circulating monocytes and alveolar macrophages, but not airway inflammation and AHR.

**Figure:**
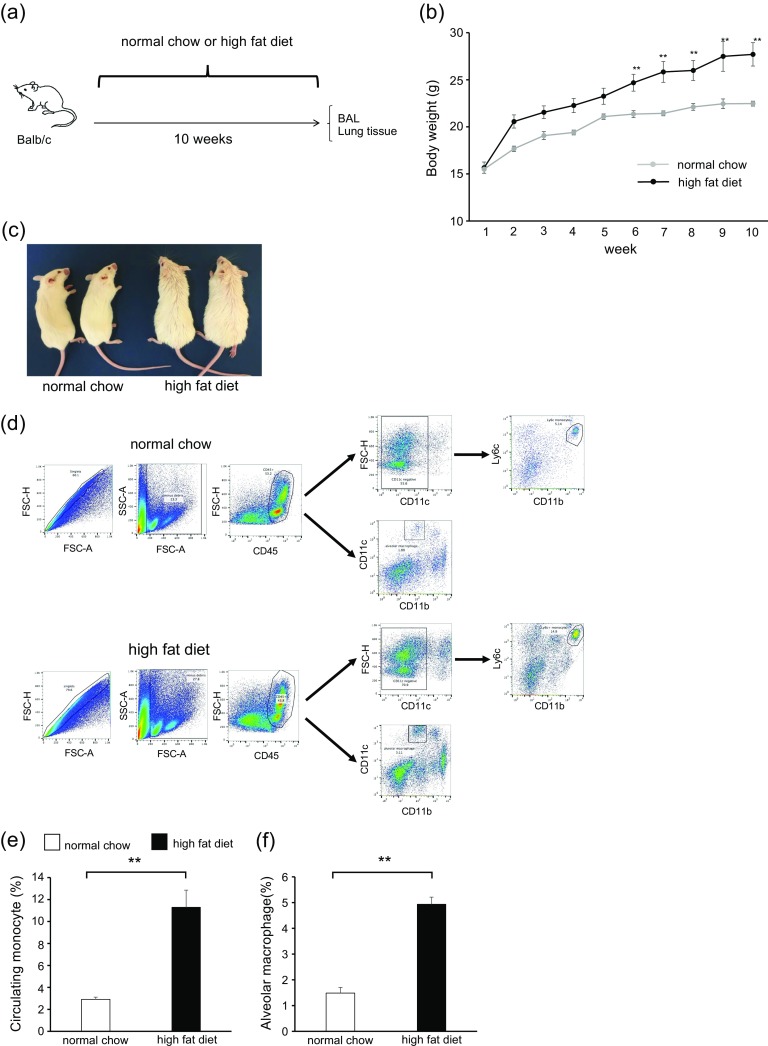

### High-Fat Diet Augmented House Dust Mite-Induced Neutrophilic Airway Inflammation, Airway Hyperresponsivenes, and Cytokine Production in the Lungs

**Fig. 2:**
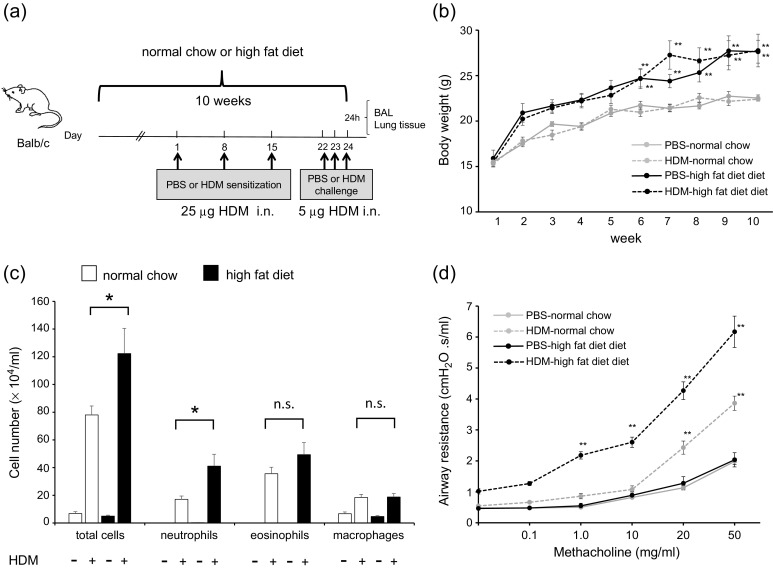

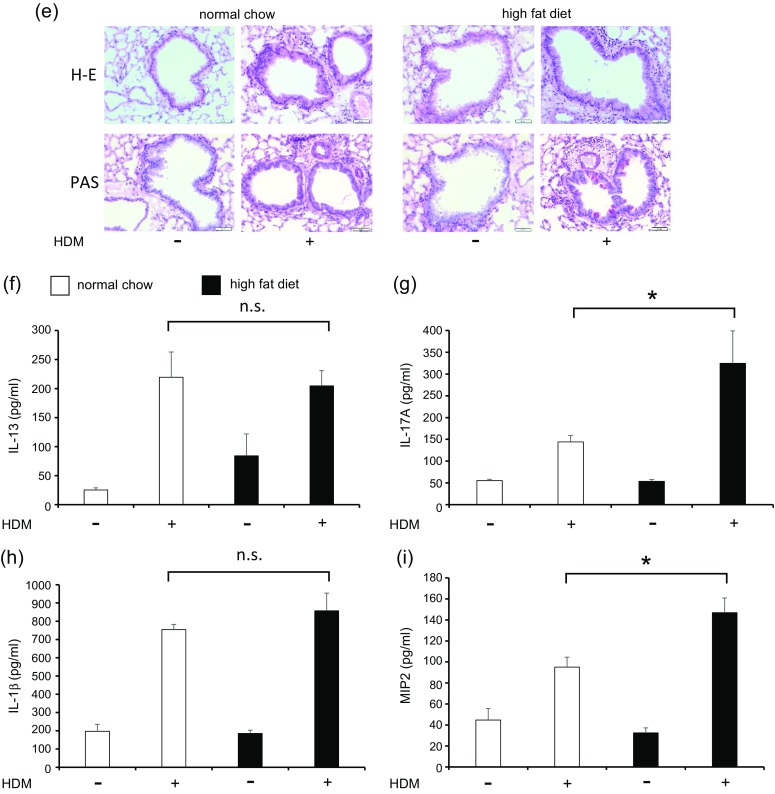
HFD augments HDM-induced neutrophilic airway inflammation, airway hyperresponsiveness, and cytokine production in the lungs. **a** Protocol of HDM-induced airway inflammation in normal chow or HFD mouse model. **b** Body weight gain is compared among PBS-chow, HDM-chow, PBS-HFD, and HDM-HFD mice (*n* = 6 in each group). **c** Bronchoalveolar lavage fluid analysis for total and differential cell counts among PBS-chow, HDM-chow, PBS-HFD, and HDM-HFD mice (*n* = 6 in each group). The HDM-HFD group is compared with the HDM-chow group. **d** Airway hyperresponsiveness is measured through assessment of airway resistance according to graded concentrations of methacholine in PBS-chow, HDM-chow, PBS-HFD, and HDM-HFD mice (*n* = 6 in each group). The HDM-chow group is compared with the PBS-chow group, while the HDM-HFD group is compared with HDM-chow mice. **e** Histologic examination for airway inflammation. Sections are stained with H & E (*upper panels*) and PAS (*lower panels*). Original magnification was × 200. Concentrations of **f** IL-13, **g** IL-17A, **h** IL-1β, and **i** MIP2 in lung tissue are measured by ELISA (*n* = 6 in each group). *P < 0.05, **P < 0.01. *HFD*, high-fat diet; *HDM*, house dust mite; *PBS*, phosphate-buffered saline; *H & E*, hematoxylin and eosin; *PAS*, periodic acid-Schiff; *MIP2*, macrophage inflammatory protein 2; *ELISA*, enzyme-linked immunosorbent assay.

We investigated the association of HFD with airway inflammation, AHR, and cytokine level in HDM-induced mice (Fig. 2a). The body weights of PBS-HFD and HDM-HFD mice were significantly higher than those of PBS-chow and HDM-chow mice (Fig. 2b). BALF total cell count, neutrophils, and eosinophils were significantly higher in HDM-chow and HDM-HFD mice than in PBS-chow and PBS-HFD mice (Fig. 2c). Additionally, among the HDM mice, the BALF total cell count and neutrophils, not eosinophils, were significantly increased in HFD mice compared to chow mice (Fig. 2c). Airway resistance, represented by AHR, was higher in HDM-chow mice than in PBS-chow and PBS-HFD mice and was significantly higher in HDM-HFD mice than in HDM-chow mice (Fig. 2d).

On pathologic examination of the lungs, inflammatory cells and goblet cell hyperplasia were intensely seen in HDM-normal chow mice and HDM-HFD mice compared with PBS-chow mice and PBS-HFD mice. The findings in HDM-HFD mice tended to be more intense than those in HDM-chow mice (Fig. 2e). The levels of cytokines IL-13, IL-17A, IL-1β, and MIP2 in lung tissue were increased in HDM-chow and HDM-HFD mice compared with PBS-chow and PBS-HFD mice. IL-17A and MIP2, but not IL-13 and IL-1β, were significantly increased in the lungs of HDM-HFD mice compared with those of HDM-chow mice. According to these data, HDM-induced neutrophilic airway inflammation, AHR, and production of IL-17A and MIP2 cytokines in the lungs were augmented by an HFD, probably by increase in the number of lung macrophages, which might be related to the progression of airway inflammation.

### Saturated Fatty Acids Induced *In Vitro* Monocyte Chemoattractant Protein-1 and Lipopolysaccharide-Primed Inflammatory Cytokine Production from Macrophages

To clarify the presence of a direct interaction between HFD and macrophages, we used *in vitro* assays of SFA and several macrophages. In an official analysis of the fatty acid profile of HFD, 37.1% comprised SFA, of which PA was the most common at 62.2%. Therefore, we used PA to represent SFA and found that it induced the expression of TLR4 and increased MCP-1 in RAW cells (Fig. 3a, b). Because HDM did not induce cytokine production from macrophages *in vitro* (data not shown), we cultured macrophages with PA and LPS instead. LPS increased the levels of TNF-α and IL-1β in RAW cells (Fig. 3c, d), BMMs (Fig. 3e, f), and peritoneal macrophages (Fig. 3g, h); these levels were significantly increased further by pre-incubation with PA. These data suggested that SFA induced TLR4 expression and macrophage recruitment and directly augmented LPS-related inflammation. SFA was considered a factor that affected airway inflammation in HDM-HFD mice.

**Figure:**
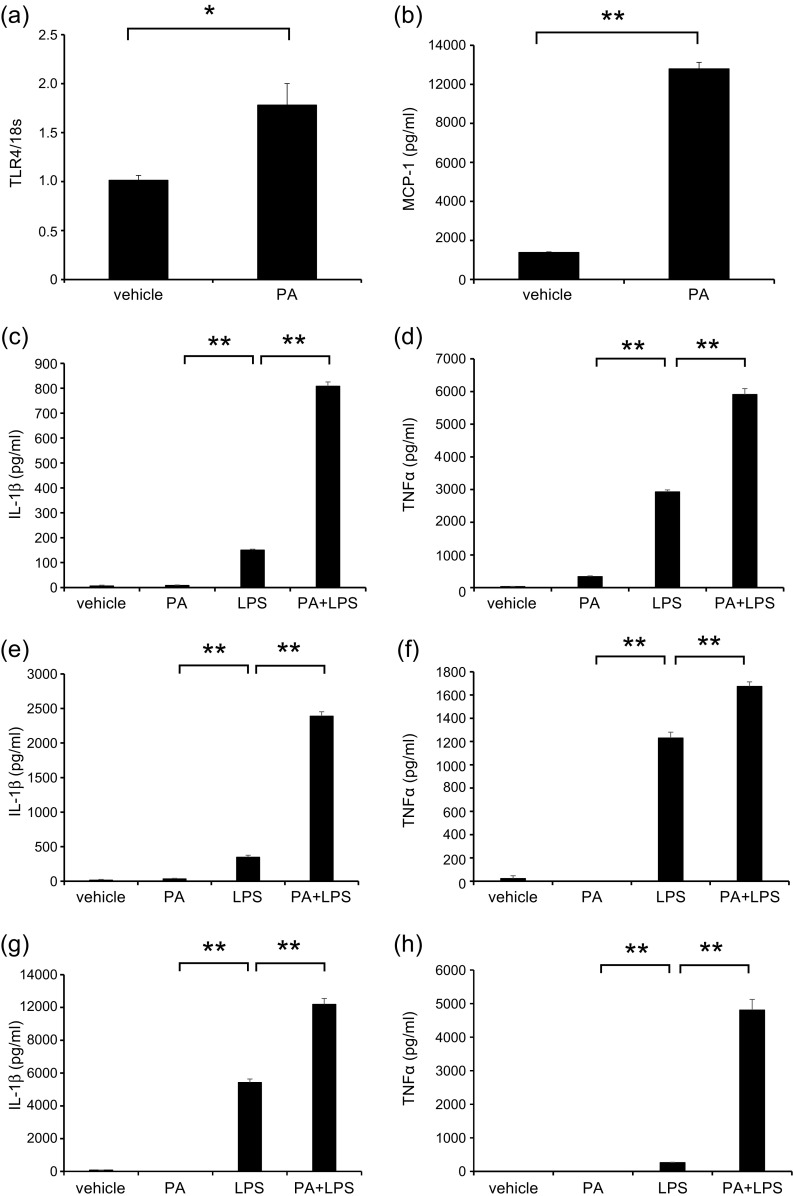

### Saturated Fatty Acid Administration Increased Lung Macrophages but Not Airway Inflammation and Airway Hyperresponsiveness in Mice

We administered PA intraperitoneally to mice [13, 26] and investigated the cell population in the lungs (Fig. 4a). The body weights of PA-administered mice and vehicle mice were not different (data not shown). Circulating monocytes and alveolar macrophages were significantly increased in the lungs of PA-administered mice compared with those in vehicle mice (Fig. 4b–d). BALF total cell count and differential count, as well as AHR, were not different between PA-administered mice and vehicle mice (data not shown). These data suggested that similar to HFD, PA increased the number of lung macrophages, including circulating monocytes and alveolar macrophages, without airway inflammation and AHR.

**Figure:**
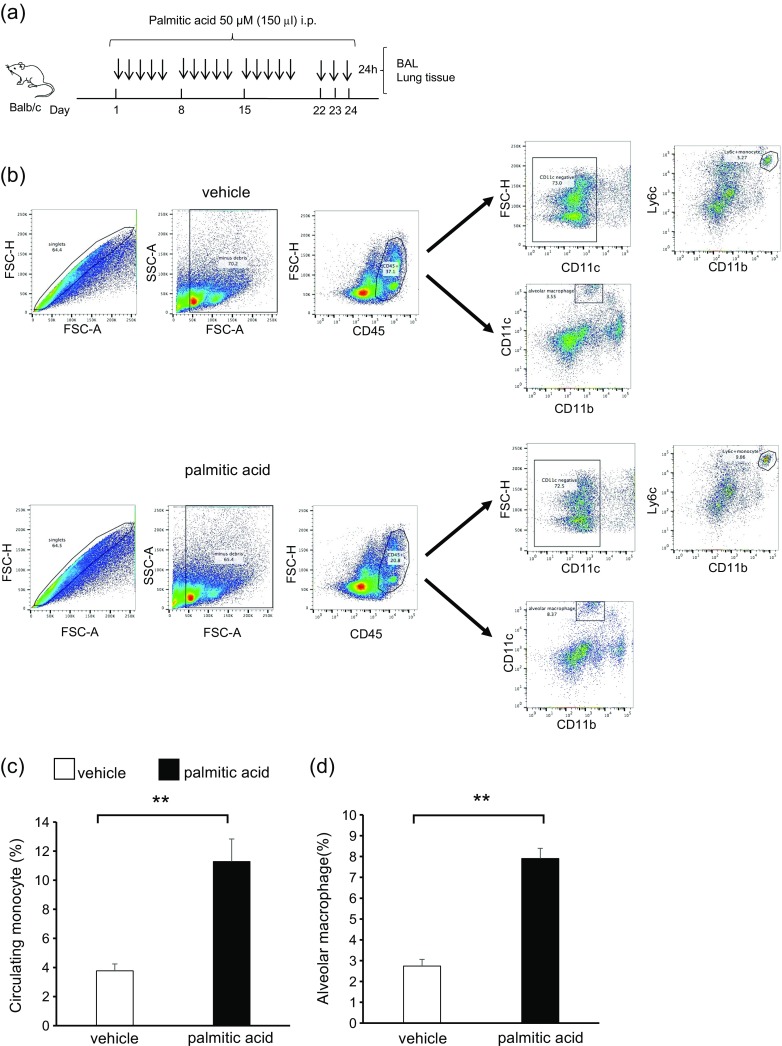

### Saturated Fatty Acids Augmented House Dust Mite-Induced Neutrophilic Inflammation, Airway Hyperresponsiveness, and Cytokine Levels in the Lungs

**Fig. 5:**
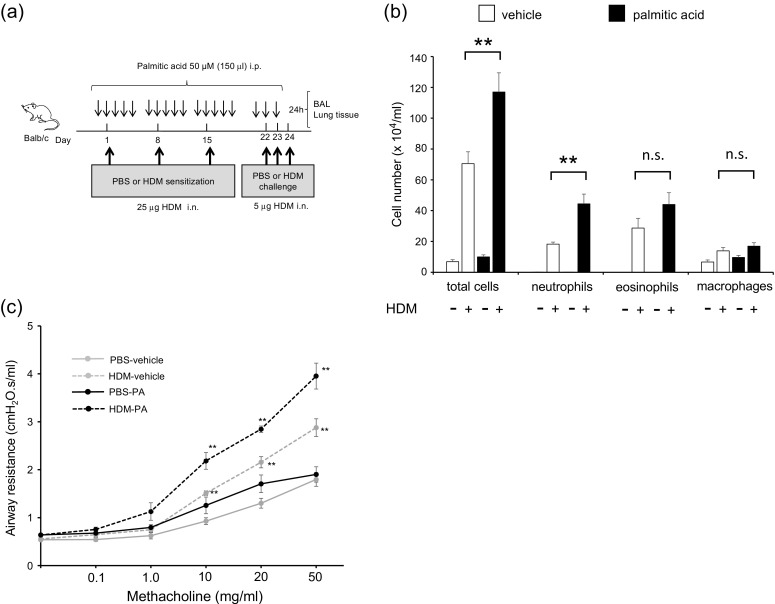

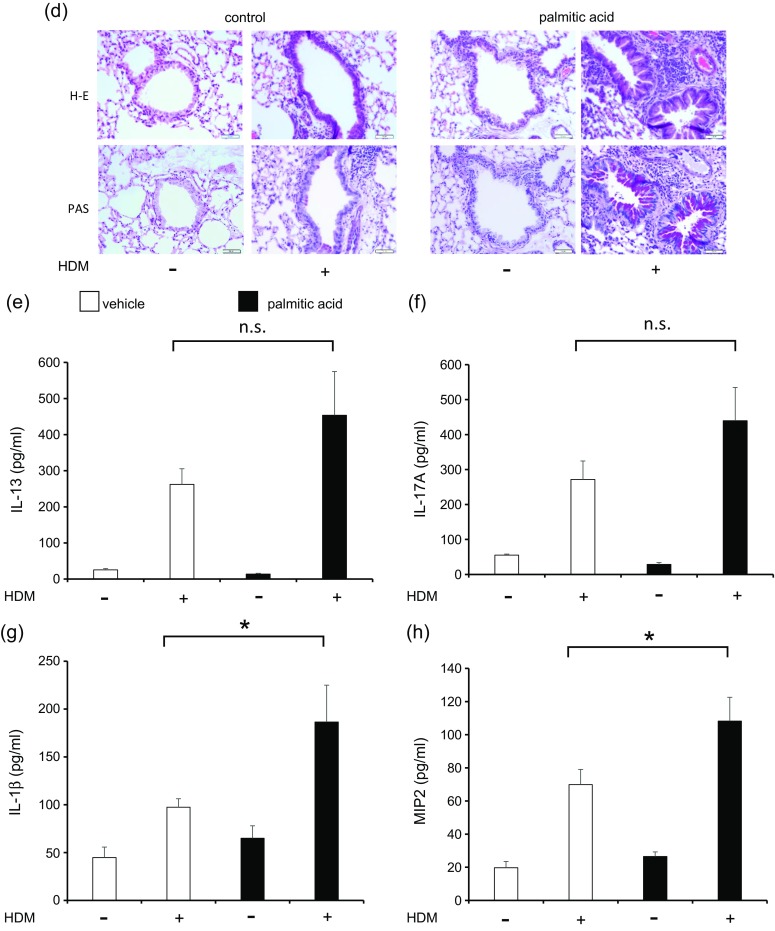
PA arguments HDM-induced neutrophilic airway inflammation, airway hyperresponsiveness, and cytokine production in the lungs. **a** Protocol of HDM-induced airway inflammation mouse model administered with vehicle or PA. **b** Bronchoalveolar lavage fluid total and differential cell counts is compared among PBS-vehicle, HDM-vehicle, PBS-PA, and HDM-PA mice (*n* = 6 in each group). The HDM-vehicle group is compared with the HDM-PA group. **c** Airway hyperreactivity is assessed by measuring airway resistance according to graded concentrations of methacholine in PBS-vehicle, HDM-vehicle, PBS-PA, and HDM-PA mice (*n* = 6 in each group). The HDM-vehicle group is compared with the PBS-vehicle group, while the HDM-PA group is compared with the HDM-vehicle mice. **d** Histologic examination for airway inflammation. Sections are stained with H & E (*upper panels*) and PAS (*lower panels*). Original magnification was ×200. Concentrations of **e** IL-13, **f** IL-17A, **g** IL-1β, and **h** MIP2 in the lung tissue were measured by ELISA (*n* = 6 in each group). *P < 0.05, **P < 0.01. *HDM*, house dust mite; *PA*, palmitic acid; *PBS*, phosphate-buffered saline; *H & E*, hematoxylin and eosin; *PAS*, periodic acid-Schiff; *MIP2*, macrophage inflammatory protein 2; *ELISA*, enzyme-linked immunosorbent assay.

Finally, we sensitized and exposed PA-administered mice with HDM to identify the role of SFA in the pathogenesis of asthma (Fig. 5a). Body weight did not differ among PBS-vehicle mice, HDM-vehicle mice, PBS-PA mice, and HDM-PA mice (data not shown). BALF total cell count, neutrophils, and eosinophils were significantly higher in HDM-vehicle and HDM-PA mice than in PBS-vehicle mice and PBS-PA mice (Fig. 5b). Total cell count and neutrophils were significantly increased in the BALF of HDM-PA mice compared with those in HDM-vehicle mice. Eosinophils tended to be increased in HDM-PA mice than in HDM-vehicle mice; however, this was not significantly different (Fig. 5b). Airway resistance was higher in HDM-vehicle mice than in PBS mice and further increased significantly in HDM-PA-administered mice than in HDM-vehicle mice (Fig. 5c).

On pathologic examination of the lungs, the presence of inflammatory cells and goblet cell hyperplasia was more intense in HDM-vehicle mice and HDM-PA-administered mice than in PBS-vehicle mice and PBS-PA-administered mice. The findings in HDM-PA-administered mice tended to be more intense than those in HDM-control mice (Fig. 5d). IL-13, IL-17A, IL-1β, and MIP2 cytokines in lung tissue were increased in HDM-vehicle mice and HDM-PA-administered mice compared to PBS-vehicle mice and PBS-PA-administered mice. IL-13 and IL-17A tended to be higher in the lungs of HDM-PA-administered mice than in HDM-vehicle mice, but this was not statistically significant (Fig. 5e, f). IL-1β and MIP2 significantly increased in the lungs of HDM-PA-administered mice compared to HDM-vehicle mice (Fig. 5g, h).

## DISCUSSION

The present study demonstrated that SFA had important roles in the augmenting the mechanisms of asthma in obesity. Specifically, these roles included progression of neutrophilic airway inflammation and AHR. HFD, which comprised a large amount of SFA, increased the number of macrophages in the lungs and exacerbated neutrophilic airway inflammation and AHR. This observation was associated with elevation in the levels of IL-17A and MIP2 cytokines in the lungs. Moreover, intraperitoneal administration of SFA showed similar effects with HFD mice in increasing lung macrophages and progression of HDM-induced neutrophilic airway inflammation and AHR, along with increased IL-1β and MIP2 cytokines in the lungs. PA affected not only MCP-1 induction but also TNF-α and IL-1β production and TLR4 upregulation in macrophages. To the best of our knowledge, this was the first report that clarified the augmenting mechanisms of asthma in obesity in relation to SFA and macrophages.

In the present study, lung macrophages, including circulating monocytes and alveolar macrophages, were significantly increased in HFD mice and PA-administered mice, although there was no difference in the number of macrophages in BALF. A previous study reported that the phenotypes of alveolar and interstitial macrophages in BALF were different from those in lung tissue [51]. We considered that interstitial macrophages might be related to the increasing number of macrophages in lung tissue. Macrophages were reported as pivotal regulators of immunity and inflammation in obesity and asthma [10, 32]. Mcneils et al. reported that obesity increased the recruitment of tissue macrophages and led to inflammation in adipose tissue, liver, and skeletal muscle [25]. Obesity has been shown to regulate macrophage phenotype and to induce inflammatory signals, such as nuclear factor-κB and phosphatidylinositol 3-kinase [6, 49]. In addition, SFA was shown to modulate TNF-α expression in mice macrophage lineage and to activate inflammation through nucleotide-binding domain, leucine-rich repeats containing family, and pyrin domain-containing-3 inflammasome [9, 44]. Circulating and alveolar macrophages are crucial for airway inflammation and are related to the pathogenesis of severe asthma through LPS responsiveness [19, 31]. We have reported that circulating macrophages, as the source of IL-33, contributed to severe asthma [42]. The findings in this study of increased lung macrophages in HFD mice and PA-administered mice were consistent with those of previous studies.

In the present study, neutrophilic airway inflammation and AHR were augmented along with elevation of MIP2 and IL-17A in the lungs. A previous study reported that asthmatic patients with obesity (BMI >30) had poor asthma-specific quality of life, poor asthma control, and frequent asthma-related hospitalizations, compared with non-obese (BMI <25) asthma patients [29]. Recent studies showed that innate lymphoid cell 3-induced IL-17 production was related to obesity-associated AHR through macrophage-derived IL-1β [22] and that blockade of TNF-α attenuated ozone-induced neutrophilic inflammation and AHR in obesity [48]. Additionally, PA induced islet inflammation and recruited macrophages with MCP-1 through TLR4 *in vivo* [13]. According to these data, we considered that SFA recruited macrophages to the lungs and caused progression of HDM-induced neutrophilic airway inflammation in refractory asthma. This ICS-insensitive phenotype might be a result of IL-17A and MIP2 induction, as demonstrated in this HFD mouse model.

We have shown that PA induced MCP-1 production and exacerbation of LPS-primed inflammatory cytokine production from macrophages. MCP-1 was reported as an obesity-related chemokine that modulates tissue migration of macrophages [30]. In addition, obese patients were shown to express higher plasma levels of MCP-1 than normal patients [5, 7]. LPS, which is contained in HDM, caused a shift from eosinophilic to neutrophilic airway inflammation, along with elevation of IL-8; these contributed to resistance to asthma treatment [24, 53]. According to these results, attenuation of SFA levels might control neutrophilic airway inflammation in obese patients with asthma. In the future, reduction of SFA-regulated migration of lung macrophages may be a target of treatment in severe asthma patients with obesity.

In conclusion, SFA induced MCP-1 production for macrophage recruitment to the lungs and directly enhanced LPS-induced TNF-α and IL-1β production by macrophages. Furthermore, SFA increased the number of lung macrophages, augmented HDM-induced neutrophilic airway inflammation and AHR, and increased the levels of IL-17A and MIP2 in HFD mice.
